# Primary myelofibrosis and the "bad seeds in bad soil" concept

**DOI:** 10.1186/1755-1536-5-S1-S20

**Published:** 2012-06-06

**Authors:** Marie-Caroline Le Bousse-Kerdilès

**Affiliations:** 1 The French INSERM and the European EUMNET networks on Myelofibrosis, The French Intergroup of Myeloproliferative disorders (FIM), INSERM U972, Paris XI University, Laboratory of Hematology, Paul Brousse Hospital, 14, av. Paul-Vaillant Couturier ; 948007, Villejuif Cedex, France

## Abstract

Primary Myelofibrosis (PMF) is a chronic myeloproliferative neoplasm characterized by a clonal myeloproliferation and a myelofibrosis. The concomitant presence of neoangiogenesis and osteosclerosis suggests a deregulation of medullar stem cell niches in which hematopoietic stem cells are engaged in a constant crosstalk with their stromal environment. Despite the recently discovered mutations including the JAK2^Val617F ^mutation, the primitive molecular event responsible for the clonal hematopoietic proliferation is still unknown. We propose that the "specificity" of the pathological process that caracterizes PMF results from alterations in the cross talk between hematopoietic and stromal cells. These alterations contribute in creating a abnormal microenvironment that participates in the maintenance of the neoplasic clone leading to a misbalance disfavouring normal hematopoiesis; in return or simultaneously, stromal cells constituting the niches are modulated by hematopoietic cells resulting in stroma dysfunctions. Therefore, PMF is a remarkable "model" in which deregulation of the stem cell niche is of utmost importance for the disease development. A better understanding of the crosstalk between stem cells and their niches should imply new therapeutic strategies targeting not only intrinsic defects in stem cells but also regulatory niche-derived signals and, consequently, hematopoietic cell proliferation.

## Introduction

Philadelphia-negative chronic myeloproliferative neoplams (Ph^- ^MPNs) are clonal hemopathies that arise from the oncogenic transformation of hematopoietic stem/progenitor cells that conserve full differentiation potential with qualitative and quantitative abnormalities [1]. They share several common features in including abnormal proliferation of hematopoietic cells from one or more cell lineages with a hypersensitivity to growth factors and decreased apoptosis [2]. In contrast to chronic myeloid leukaemia, the molecular mechanisms leading to Ph^- ^MPN progression have remained unclear until the recent finding of the V617F JAK2 mutation in most of Polycythemia Vera (PV) and half of Essential Thrombocytosis (ET) and Primary Myelofibrosis (PMF) cases ([3], see for review [4]). This discovery has revolutionized the understanding of the biology of at least PV, by showing that kinase pathway alterations are part of the pathological process leading to hematopoietic proliferation and growth factor hypersensitivity. However, the fact that the JAK2^+ ^syndromes exhibit various clinical features raises the question of how a single mutation can generate different MPNs and strongly suggests that other acquired events have to occur, at least in ET and PMF.

**Figure 1:**
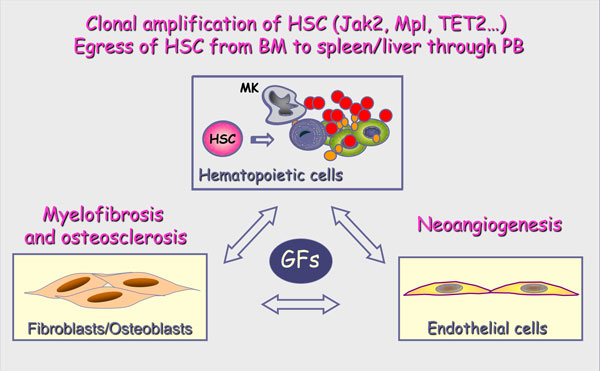
**Pathophysiological characteristics of PMF**. Primary myelofibrosis is characterized by a clonal amplification of hematopoietic stem cells (HSCs) and a prominent proliferation of "dystrophic" megakaryocytes (MK) that partly result from the presence of gain-of-function mutations involving JAK2 and MPL genes and that is associated with a migration of HSC from bone marrow (BM) to spleen and liver through peripheral blood (PB). Such myeloproliferation is associated with alterations of stroma featured by a myelofibrosis, an osteosclerosis and a neoangiogenesis. This stromal reaction is reported to be secondary to the stimulation of stromal cells including fibroblasts, osteoblasts and endothelial cells by growth factors (GFs) produced in excess by cells from the hematopoietic clone and especially by MK cells.

Besides the potential role of altered growth factor signaling pathways, a number of converging arguments suggests that changes within the hematopoietic environment also take part in MPN pathogenesis. The most significant elements in favor of this assumption emerge from studies on PMF that is the rarest and most complex Ph^- ^MPN. Primary myelofibrosis is characterized by an extramedullary hematopoiesis with progressive hepato-splenomegaly resulting from the prominent mobilization of hematopoietic progenitors from bone marrow to spleen and liver. Such alteration of hematopoiesis is constantly associated with profound modifications of the bone marrow and spleen stroma as demonstrated by the presence of myelofibrosis, osteosclerosis and neoangiogenesis in PMF patients [5] (Figure 1).

Recently, evidences are accumulating that stromal cells may play an active role in the promotion or maintenance of myeloproliferative disorders in mice and that stromal dysfunction can even act as a primary enabler of inefficient hematopoiesis and secondary hematopoietic neoplastic transformation [6,7]. In human MPNs and especially in PMF, we hypothesised that alterations of stromal cells contribute to the hematopoietic clone development [8], therefore revisiting the "good seeds (stem cells) in bad soil (stroma)" model [9] in the "bad seeds in bad soil" concept.

## Hematopoiesis is regulated by medullar niches

According to A. Spradling, "the true nature of stem cells can be learned only by discovering how they are regulated" [10]. In the bone marrow, the proliferation and differentiation of hematopoietic stem cells (HSC) are under control of cellular and humoral regulatory signals created by the hematopoietic stem cell niches (see for review [11]). Within these niches, HSC are engaged in a constant crosstalk with their environment, responding to numerous signals such as secreted growth factors, oxygen and calcium variations, and are maintained in close contact with stromal cells within the proximity of the endosteal surface and of the vascular network via adhesion molecules.

**Figure 2:**
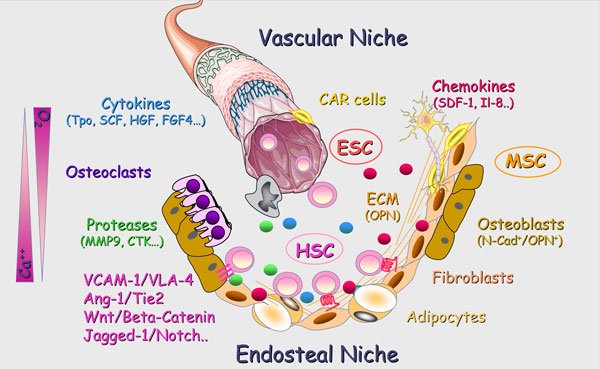
**Schematic representation of the medullar niches**. In healthy adults, hematopoiesis occurs in the bone marrow where hematopoietic stem cells (HSCs) are engaged in a constant crosstalk within specific niches. Very schematically, these haematopoietic stem cell niches are conceptually divided into two niches: The endosteal niche in which osteoblasts, derived from Mesenchymal Stromal Cell (MSC) differentiation and contributing to bone formation and osteoclats, derived from HSCs, and participating in bone resorption played a crucial role. The vascular niche in which endothelial cells, resulting from the proliferation and differentiation of Endothelial Stem Cells (ESC), are in close contact with HSC within the perivascular spaces. Beyond cell-cell contacts through receptor-ligand and adhesion molecule interactions, extracellular matrix component (EMC), cytokines and chemokines, proteases, calcium and oxygen concentrations are essential environmental components of these niches.

Whether the concept of hematopoietic niches is dating from Schofield in 1978 [12], their exact location in the bone marrow is still under debate. In healthy adult mice, it is commonly accepted that most HSCs are located close to the endosteal lining cells in the trabecular area. Staining of sections of adult hematopoietic tissues also revealed the presence of HSCs around bone marrow sinusoids (perivascular niches) (Figure 2). However, it is uncertain whether both locations represent niches and, if so, whether they are spatially distinct niches or whether endosteal cells and perivascular cells collaborate to form a common niche in which mesenchymal stem cells would be an essential link [13-15]. Furthermore, it remains to be determined whether these locations actively promote the maintenance of HSCs, or whether HSCs simply pass through some of them during their migration.

Several cellular components have been suggested to comprise the endosteal niches; these include osteoblasts, CXCL12 abundant reticular cells (CAR), osteoclasts and sympathetic neurons [13,16-18]. Dormant HSCs are probably tightly anchored in endosteal niches through interactions with numerous adhesion molecules such as N-cadherin, CD44 and various integrins. Several growth factor- and chemokine-receptors expressed by HSCs that bind soluble or membrane-bound ligands produced by niche cells have been shown to be crucial for inhibiting HSC division by retention within the niche, thus preserving their dormancy and progressive lost by exhaustion [13,14].

Anatomical relationships of HSCs with bone marrow stroma cells have also implied vascular endothelial-cadherin^+ ^sinusoidal endothelial cells (SECs) [19,20], perivascular cells and mesenchymal stem cells (MSCs) [15,21] as additional components of the niche. Both osteoblastic [16] and endothelial cells [22] can promote the maintenance of HSCs in culture, and both cell types influence each other, which makes it difficult to attribute specific functions to the endosteal or perivascular niches [23]. Moreover, a significant number of HSCs are not localized adjacent to the endosteum and sinusoids, suggesting that additional cells, including adiponectin-secreting adipocytes, may also contribute to the maintenance of HSCs *in vivo *[24].

## Primary Myelofibrosis, a disease associating a clonal myeloproliferation and an alteration of hematopoietic stroma

### The clonal myeloproliferation

In PMF patients, the myeloproliferative process is characterized by several abnormalities [9,25] including : i) the multipotency of the hematopoietic clonal stem cells with myeloid and lymphoid differentiation although an absolute lymphopenia has been described in peripheral blood, ii) the progressive dominance of clonal hematopoiesis over normal polyclonal hematopoiesis, resulting in the overproduction of one or more of the mature blood elements, iii) the hypersensitivity of hematopoietic progenitors (HP) to growth factors, iv) a striking involvement of the megakaryocytic (MK) lineage, with hyperplasia and dysplasia resulting in an excessive production of a number of cytokines and chemokines and, v) the lack of a consistent cytogenetic abnormality but the presence of mutations in the JAK2 and in the MPL thrombopoietin receptor genes.

Whereas the JAK2^Val617F ^acquired mutation represents the first reliable molecular marker of Ph^- ^MPN, it might not be the first genetic event and the molecular causes of the clonal myeloproliferation in PMF patients are still enigmatic. The pathogenic role of mutated JAK2 and more recently of mutated Mpl most likely goes through abnormal activation of signaling molecules including STAT and MAPK pathways that has been suggested to take part in the hematopoietic proliferation and increased sensitivity to cytokines [26,27]. Several other mechanisms have been proposed to participate in the dysmegakaryopoiesis, including NF-κB [28] activation, IL-8 [29] over-expression and more recently FL/Flt3 activation [27].

### The stromal reaction

**Figure 3:**
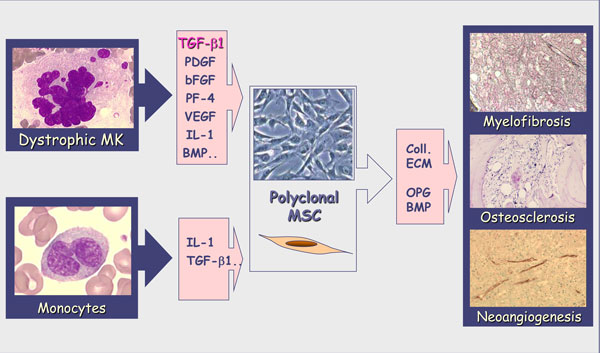
**Role of growth factors in PMF stromal reaction**. In PMF, myelofibrosis is a multifactor process resulting from alterations of fibroblasts/MSC leading to an increased deposit of extracellular matrix components resulting from growth factors released by malignant hematopoietic cells and especially MK and monocytes. These growth factors would further activate stromal cells, leading to myelofibrosis, osteosclerosis and neoangiogenesis.

**Figure 4:**
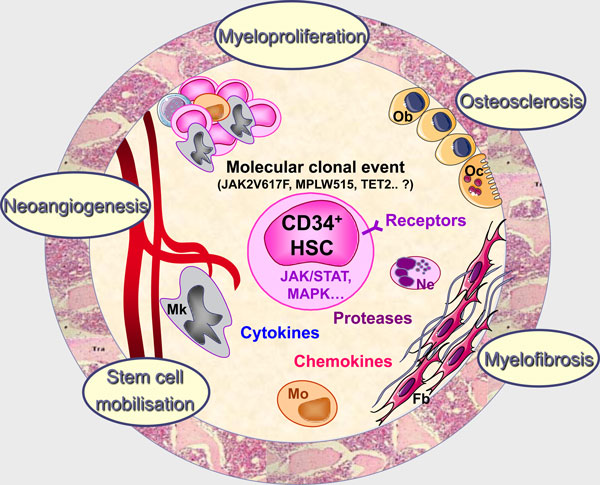
**PMF; the "Bad Seeds in Bad Soil" model**. In PMF, whereas the initial molecular defect at the origin of the hematopoietic clone is still unknown, altered interactions between CD34^+ ^HSCs and stromal cells in the medullar environment would result in an increased cytokine production by the clonal hematopoietic cells and especially by dystrophic megakaryocytes and monocytes. Production in excess of hematopoietic, fibrogenic, and angiogenic growth factors would stimulate myelofibrosis, osteosclerosis and angiogenesis through activation of stromal and endothelial cells. Consequently, by acquiring new properties, stromal cells would create a pathological microenvironment that participates in the development and maintenance of the hematopoietic clone. Mk: Megakaryocytes; Mo: Monocytes; Ne: Neutrophiles; Ob: Osteoblasts; Oc: osteoclasts; Fb: Fibroblasts.

It is accepted that myelofibrosis associating the clonal myeloproliferation is a multifactor process resulting from alterations of fibroblasts leading to the modified expression of adhesion molecules and to an increased deposit of extracellular matrix components [30]. This accumulation is suggested to be the consequence of intramedullary release of growth factors by the malignant hematopoietic clone and especially by dysplastic megakaryocytes [31]. Among these growth factors, some would further activate mesenchymal cells (PDGF, bFGF, TGFβ...), leading to the myelofibrosis, as well as endothelial cells (bFGF, VEGF, IL-8...), participating in the neoangiogenesis [32] (Figure 3). It has been hypothesized that an increased production of osteoprotegerin by stromal and endothelial cells contributed to the unbalanced osteoblast production leading to the osteosclerosis frequently associated with myelofibrosis and to vascular complications [33]. The abnormal trafficking of CD34^+ ^hematopoietic progenitors and endothelial precursors that features PMF is likely resulting from modification of their adherence to the bone marrow stroma allowing them to escape from this niche into the circulation with homing to the spleen and liver. Several mechanisms, including disturbance of CXCL12/SDF-1α-CXCR4 signal [34] through a hypermethylation of its CXCR4 receptor resulting in a down-regulation of its expression [35], and an increased extracellular matrix proteolytic activity [36], also participate in this migration. Thus, in PMF, whereas the primitive molecular event is still unknown, the "specificity" of the pathological process would result from alterations in the cross talk between hematopoietic and stromal cells. In this process, stromal cells are conditioned by growth factor produced by malignant hematopoietic cells and reciprocally, by acquiring new properties, stromal cells create a pathological microenvironment that takes part in the development and maintenance of the clone, leading to an imbalance that compromises normal hematopoiesis (Figure 4). Therefore, ignoring the environmental cues that control HSC fate during homeostasis, neoplastic HSCs can survive at anatomical sites (i.e., spleen and liver) unable to support normal adult hematopoiesis [8].

### Primary Myelofibrosis and the "bad seeds in bad soil" concept

**Figure 5:**
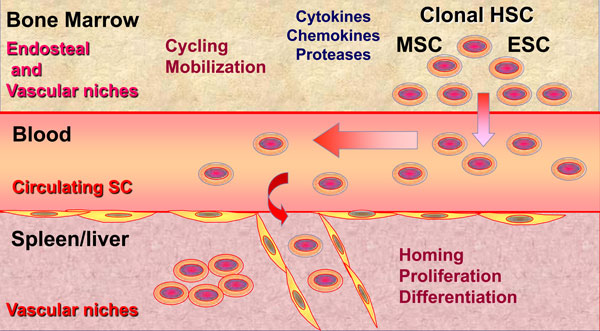
**PMF stem cells moving from niches to niches**. In response to several environmental factors, an imbalance between endosteal and vascular niches within the bone marrow would favor the proliferation and mobilization of pathological stem cells from the bone marrow to the blood. These stem cells would migrate into the spleen/liver where newly created or reinitialized vascular niches would favor their homing and differentiation resulting in an extramedullary hematopoiesis in these organs.

In PMF, it could be suggested that in response to several environmental factors including cytokines, chemokines, proteases, adhesion molecules, alteration of calcium and oxygen concentration... there is an imbalance between endosteal and vascular niches within the bone marrow. Such disequilibrium would favor the proliferation and mobilization of pathological stem cells including hematopoietic, mesenchymal and endothelial stem/progenitor cells from the bone marrow to the blood, leading to bone marrow aplasia and stem cell mobilization. These stem cells would then migrate into the spleen where newly created or reinitialized vascular niches would favor their homing and abnormal differentiation resulting in an extramedullary hematopoiesis at the origin of the splenomegaly that features this disease [8] (Figure 5). Therefore, by contributing to the hematopoietic clone development, alterations of stromal cells within hematopoietic stem cell niches are key actors of PMF pathogenesis process illustrating the "Bad Seeds in Bad Soil" concept that we proposed (Figure 4).

While chromosomal abnormalities have not been described in stromal fibroblasts, it could be questioned whether the initial oncogenic event leading to the MPN development might occur in a primitive mesodermal stem cell. The discovery, in patients with chronic myeloid leukemia, of bi-potent hemato-endothelial stem cells which harbor the Ph translocation supports such hypothesis. In PMF, while the clonality of hematopoietic stem cells is clear, the contribution of mesenchymal and/or endothelial stem cells (participating in the hematopoietic niche) to the malignant clone is not obvious and is still a matter of debate [37,38]. However, till now, the absence of recurrent genomic abnormalities in PMF patients does not allow us to definitively conclude on this challenging concern.

## Conclusion and perspectives

Therefore, by combining a clonal proliferation and a mobilization of hematopoietic stem cell(s) with marked alterations of the bone marrow and spleen stroma, PMF illustrates a unique model in which a "hematopoietic stem cell niche" deregulation plays a key role in the myeloproliferative process. In this manuscript, we have reviewed clinical parameters and key experimental results showing that an unbalanced between endosteal and vascular niches in bone marrow and spleen participates in the development and maintain of the clonal hematopoietic stem cell proliferation leading to this myeloproliferative syndrome. Whereas progress in the understanding of the role of hematopoietic microenvironment in PMF is obvious, a number of concerns still remain to be addressed. Among them: Why is bone marrow stem cell homing changed? Are spleen niches newly created or reinitialized and how is spleen stem cell homing developed? Why are bone marrow/endosteal niches disadvantaged to the benefit of spleen/endothelial niches? Do PMF HSCs exhibit different sensitivity with respect to the two types of niches and if so, is there a role for the JAK2 and MPL mutations in this altered sensitivity? Are there correlations between clinical phenotype and alterations of the niches? Are niche-initiating stem cells (MSCs) mobilized?...

Besides the conceptual importance of these issues, new insights into the possible role of hematopoietic niche deregulation in the pathogenesis of PMF should open innovative therapeutic strategies for patients whose treatment has been largely palliative until now. Of course, innovative therapies must target HSC and treatments with JAK2 inhibitors are currently under clinical trials with remarkable results on spleen size and constitutional symptom reduction. However, the lack of benefits on stromal alteration including myelofibrosis and on anemia in anti-JAK2 treated patients argues in favor of the need for therapies also targeting hematopoietic niches. These niche-targeted drugs would manipulate the competitive balance between endosteal and vascular niches and therefore would modify the stem cell trafficking and homing. By forcing specific niches to reassume a normal function, such treatments would limit the proliferation and dissemination of the malignant clone. Therefore, therapeutic approaches based on associating drugs acting on the stem cell clone and on their regulatory niches might be promising!

## Abbreviations

PMF: Primary Myelofibrosis; Ph^- ^MPNs: Philadelphia-negative chronic MyeloProliferative Neoplasms; PV: Polycythemia Vera; ET: Essential Thrombocytosis; HSC: Hematopoietic Stem Cell; CAR: CXCL12 Abundant Reticular Cell; SEC: Sinusoidal Endothelial Cell; MSC: Mesenchymal Stem Cell; HP: Hematopoietic Progenitor; MK: Megakaryocyte; GF: Growth Factor; SC: Stem cell; EMC: Extracellular Matrix Component; Mo: Monocytes; Ne: Neutrophiles; Ob: Osteoblasts; Oc: osteoclasts; Fb: Fibroblasts.

## Competing interests

The author declares that they have no competing interests.
